# Supplementary material for: Clinical Trials of Stem Cell Therapy in Japan: The Decade of Progress under the National Program
Source: J Clin Med. 2022 Nov 28;11(23):7030. doi: 10.3390/jcm11237030 (PMC9736364; doi:10.3390/jcm11237030)
Supplement: Supplementary file 1 [file jcm-11-07030-s001.zip › jcm-2021891-supplementary.pdf]

Supplementary Table S1. List of clinical trials and their websites (accessed on 15 November 2022).

No. in Table 2	Project Title (Registration ID)	URL
**Projects with human somatic stem cells**		
1-1	Healing acceleration of repaired meniscus by synovial stem cells "Clinical study to assess the safety and efficacy of transplantation of autologous synovial mesenchymal stem cells in patients with knee meniscal tear" (UMIN000011881)	https://center6.umin.ac.jp/cgi-open-bin/ctr_e/ctr_view.cgi?recptno=R000013889
1-2	Enhancement of healing for extruded injured meniscus by transplantation of synovial stem cells after meniscus surgery (UMIN000017890)	https://center6.umin.ac.jp/cgi-open-bin/ctr_e/ctr_view.cgi?recptno=R000020728
1-3	Investigator initiated clinical trial of autologous synovial stem cells for meniscus lesions (UMIN000026383)	https://center6.umin.ac.jp/cgi-open-bin/ctr_e/ctr_view.cgi?recptno=R000030110
1-4	Intraarticular injections of synovial stem cells for osteoarthritis of the knee (UMIN000026732)	https://center6.umin.ac.jp/cgi-open-bin/ctr_e/ctr_view.cgi?recptno=R000029967
2-1	Clinical application of corneal endothelial regenerative medicine by means of cultured human corneal endothelial cell transplantation (UMIN000012534)	https://center6.umin.ac.jp/cgi-open-bin/ctr_e/ctr_view.cgi?recptno=R000014592
2-2	Investigator initiated trial of cultivated human corneal endothelial cell injection (UMIN000028324)	https://center6.umin.ac.jp/cgi-open-bin/ctr_e/ctr_view.cgi?recptno=R000032417
2-3	Investigator initiated confirmatory trial of cultivated human corneal endothelial cell injection (UMIN000034334)	https://center6.umin.ac.jp/cgi-open-bin/ctr_e/ctr_view.cgi?recptno=R000039139
2-4	Clinical application of corneal endothelial regenerative medicine by means of cultured human corneal endothelial cell transplantation: long-term follow up (UMIN000036422)	https://center6.umin.ac.jp/cgi-open-bin/ctr_e/ctr_view.cgi?recptno=R000041501
3-1	Safety study of a less invasive liver regeneration therapy using cultured autologous bone marrow-derived mesenchymal stem cells for decompensated liver cirrhotic patient (UMIN000016686)	https://center6.umin.ac.jp/cgi-open-bin/ctr_e/ctr_view.cgi?recptno=R000019363
3-2	Study on the safety of hepatic arterial infusion of cultured autologous bone marrow cells in patients with decompensated liver cirrhosis (UMIN000035528)	https://center6.umin.ac.jp/cgi-open-bin/ctr_e/ctr_view.cgi?recptno=R000040478
3-3	An open-label, uncontrolled study to evaluate the efficacy and safety of autologous bone marrow mesenchymal stem cells (LS-ABMSC1) in patients with decompensated liver cirrhosis (Phase I/II study) (UMIN000041461)	https://center6.umin.ac.jp/cgi-open-bin/ctr_e/ctr_view.cgi?recptno=R000047329
4	Investigator-initiated clinical trial to evaluate the safety and efficacy of AX-1911, magnetic field generator for magnetic targeting, in knee osteoarthritis (jRCT2062210039)	https://jrct.niph.go.jp/en-latest-detail/jRCT2062210039
5	Mucosal regeneration therapy by autologous intestinal stem cell transplantation to inflammatory bowel disease patients (UMIN000030117)	https://center6.umin.ac.jp/cgi-open-bin/ctr_e/ctr_view.cgi?recptno=R000033766

Projects with human iPS or ES cells

6-1	A study of transplantation of autologous induce d pluripotent stem cell (iPSC) derived retinal pigment epithelium (RPE) cell sheet in subjects with exudative age related macular degeneration (UMIN000011929)	https://center6.umin.ac.jp/cgi-open-bin/ctr_e/ctr_view.cgi?recptno=R000013279
6-2	A study of transplantation of allogenic induced pluripotent stem cell (iPSC) derived retinal pigment epithelium (RPE) cell suspension in subjects with neovascular age related macular degeneration (UMIN000026003)	https://center6.umin.ac.jp/cgi-open-bin/ctr_e/ctr_view.cgi?recptno=R000029894
7	First-in-human clinical research of iPS derived corneal epithelial cell sheet transplantation for patients with limbal stem-cell deficiency (UMIN000036539)	https://center6.umin.ac.jp/cgi-open-bin/ctr_e/ctr_view.cgi?recptno=R000041628
8	A prospective observational study of induced pluripotent stem cell-derived cardiac spheres transplantation EXTENDed Follow-up (UMIN000047335)	https://center6.umin.ac.jp/cgi-open-bin/ctr_e/ctr_view.cgi?recptno=R000053954
9	Kyoto trial to evaluate the safety and efficacy of iPSC-derived dopaminergic progenitors in the treatment of Parkinson's disease (UMIN000033564)	https://center6.umin.ac.jp/cgi-open-bin/ctr_e/ctr_view.cgi?recptno=R000038278
10	Clinical study of HAES transplantation in patients with neonatal onset urea cycle disorder (JMA-IIA00412)	https://dbcentre3.jmacct.med.or.jp/jmactr/default.aspx?JMACCTID=JMA-IIA00412
11	Clinical study of autologous transfusion of iPS cell-derived platelets for thrombocytopenia (iPLAT1) (jRCTa050190117)	https://jrct.niph.go.jp/en-latest-detail/jRCTa050190117
12	Regenerative medicine for spinal cord injury at subacute stage using human induced pluripotent stem cell-derived neural stem/progenitor cells (UMIN000035074)	https://center6.umin.ac.jp/cgi-open-bin/ctr_e/ctr_view.cgi?recptno=R000039960
14-1	Clinical trial of human (allogeneic) iPS cell-derived cardiomyocytes sheet for ischemic cardiomyopathy (jRCT2053190081)	https://jrct.niph.go.jp/en-latest-detail/jRCT2053190081
14-2	Clinical trial of human (allogeneic) iPS cell-derived cardiomyocytes sheet for ischemic cardiomyopathy (Follow-up trial) (jRCT2053220055)	https://jrct.niph.go.jp/en-latest-detail/jRCT2053220055
16	A Phase I study of iPS-NKT cell intra-arterial infusion therapy in patients with recurrent or advanced head and neck cancer (First in human study) (jRCT2033200116)	https://jrct.niph.go.jp/en-latest-detail/jRCT2033200116
17	A clinical study for treatment of articular cartilage damage in knee joints with allogeneic induced pluripotent stem (iPS) cell-derived cartilage (TACK-iPS) (jRCTa050190104)	https://jrct.niph.go.jp/en-latest-detail/jRCTa050190104
